# Supplementary material for: Sex difference in parental risk of suicide attempt during and after pregnancy in Sweden
Source: Nat Hum Behav. 2025 Sep 25;10(1):101–10. doi: 10.1038/s41562-025-02311-5 (PMC12846918; doi:10.1038/s41562-025-02311-5)
Supplement: Supplementary file 1 — Supplementary Methods and Figs. 1–13. [file 41562_2025_2311_MOESM1_ESM.pdf]

nature human behaviour

Supplementary information https://doi.org/10.1038/s41562-025-02311-5

Sex difference in parental risk of suicide attempt during and after pregnancy in Sweden

In the format provided by the authors and unedited

Table of Contents

eMethods 2

Figure S1 Flow chart 15

Figure S2 Incidence rate of parental suicide attempt before, during, and after pregnancy, by age at start of each period 16

Figure S3 Standardized incidence rate of parental suicide attempt before, during, and after pregnancy, by time-varying depressive disorders 17

Figure S4 Standardized incidence rate of parental suicide attempt before, during, and after pregnancy, by history of suicide attempt 18

Figure S5 Standardized incidence rate of parental suicide attempt before, during, and after pregnancy, by week and time-varying depressive disorders 19

Figure S6 Standardized incidence rate of parental suicide attempt before, during, and after pregnancy, by week and history of suicide attempt 20

Figure S7 Incidence rate ratio of parental suicide attempt during and after pregnancy when comparing with the corresponding week before pregnancy, by time-varying depressive disorders 21

Figure S8 Incidence rate ratio of parental suicide attempt during and after pregnancy when comparing with the corresponding week before pregnancy, by history of suicide attempt 22

Figure S9 Incidence rate ratio of suicide attempt among mothers compared to the corresponding week among fathers, by suicidal method 23

Figure S10 Incidence rate ratio of parental suicide attempt during and after pregnancy when compared with average incidence rate before pregnancy 24

Figure S11 Standardized incidence rate of parental suicide attempt before, during, and after pregnancy by week, after excluding week 53 from the analysis 25

Figure S12 Incidence rate ratio of parental suicide attempt during and after pregnancy when compared with the corresponding week before pregnancy, after excluding week 53 from the analysis 26

Figure S13 Incidence rate ratio of suicide attempt among mothers when compared with the corresponding week among fathers in preconception and postpartum period, after excluding week 53 27

eMethods

Imputation of delivery date

The imputation method was developed based on an independent register-based database, with complete information on admission date, discharge date, and delivery date for all births during 2014-2021 in Sweden. If the length of the hospital stay was within 2 days, the admission date was used as the date of delivery. If the length of stay was greater or equal to 3 days, the date of delivery was imputed based on parity and mode of delivery by using the date of admission plus the median length of stay from the independent database. Using this imputation strategy, 99% of the pregnancies had imputed delivery date within ± 2 days of the true delivery date. Applying a more strict requirement for accuracy, 97% of the pregnancies had imputed delivery date within ± 1 days within the true delivery date; and 69% of the pregnancies had imputed delivery date exactly at the true delivery date.

Poisson regression

Variables in the dataset:

Variable name	Description	Level
prd	Period	0, preconception; 1, antepartum; 2, postpartum
mot	Mother indicator	0, father; 1, mother
wksp1	Spline on week	
wksp2	Spline on week	
wksp3	Spline on week	
agec	Age group	0, 11-19; 1, 20-24; 2, 25-29; 3, 30-34; 4, 35-39; 5,≥40
cal	Calendar year, group	0, 2001-2005; 1, 2006-2010; 2, 2011-2015; 3, 2016-2021
edu	Educational level (years)	1, < 10; 2, 10-12; 3, ≥ 13; 9, unknown
marr	Civil status	1, cohabitating; 2, non-cohabitating
income	Income level	0, Quantile 1; 1, Quantile 2; 2, Quantile 3; 3, Quantile 4; 4, Quantile 5; 9, Unknown
firstpreg	Primiparous	0, No; 1, Yes
fland	Country of birth	1, Sweden; 2, Europe; 3, Other; 9, Unknown
psy	History of psychiatric disorders	0, No; 1, Depressive disorders; 2, Other psychiatric disorders
sui_his	History of suicide attempt	0, No; 1, Yes
season	Season	0, spring; 1, summer; 2-autumn; 3-winter

1) In the analysis of IRRs during and after pregnancy when compared with the corresponding week before pregnancy, the following Poisson regression was modelled among mothers and fathers separately.

$\ln(\lambda)=\beta_0+\beta_1*1.prd+ \beta_2*2.prd+ \beta_3*wksp1+ \beta_4*wksp2+\beta_5*wksp3+ \beta_6* 1.prd* wksp1+ \beta7*2.prd* wksp1+ \beta_8* 1.prd* wksp2+ \beta_9*2.prd* wksp2+ \beta_{10}* 1.prd* wksp3+ \beta_{11}*2.prd* wksp3+ \beta_{12}*1.agec+ \beta_{13}*2.agec+ \beta_{14}*3.agec+ \beta_{15}*4.agec+ \beta_{16}*5.agec+ \beta_{17}*1.cal+ \beta_{18}*2.cal+ \beta_{19}*3.cal+ \beta_{20}*2.edu+ \beta_{21}*3.edu+ \beta_{22}*9.edu+ \beta_{23}*2.marr+ \beta_{24}*1.income+ \beta_{25}*2.income+ \beta_{26}*3.income+ \beta_{27}*4.income+ \beta_{28}*9.income+ \beta_{29}*1.firstpreg+ \beta_{30}*2.fland+ \beta_{31}*3.fland+ \beta_{32}*9.fland+ \beta_{33}*1.psy+ \beta_{34}*2.psy+ \beta_{35}*1.sui\_his+ \beta_{36}*1.season+ \beta_{37}*2.season+ \beta_{38}*3.season$

Parameter results among mothers:

```
------------------------------------------------------------------------------
          _d |        IRR   Std. err.      z    P>|z|     [95% conf. interval]
-------------+----------------------------------------------------------------
         prd |
          1  |   1.053312   .0912754     0.60   0.549     .8887824    1.248298
          2  |   .1283962   .0154739   -17.03   0.000     .1013837     .162606
             |
       wksp1 |   .9792349     .00415    -4.95   0.000     .9711347    .9874027
       wksp2 |   1.043389   .0131779     3.36   0.001     1.017878     1.06954
       wksp3 |    .872136   .0364084    -3.28   0.001     .8036182    .9464958
             |
 prd#c.wksp1 |
          1  |   .8852364   .0099909   -10.80   0.000     .8658695    .9050364
          2  |    1.07695   .0109097     7.32   0.000     1.055779    1.098547
             |
 prd#c.wksp2 |
          1  |   1.319566   .0521349     7.02   0.000     1.221239    1.425809
          2  |   .8863308   .0256535    -4.17   0.000     .8374504    .9380642
             |
 prd#c.wksp3 |
          1  |   .5098203   .0622097    -5.52   0.000     .4013758    .6475646
          2  |   1.347046   .1140486     3.52   0.000     1.141077    1.590193
             |
```

agec						
20-	.6179002	.0255105	-11.66	0.000	.5698701	.6699784
25-	.393935	.0180189	-20.37	0.000	.3601554	.4308829
30-	.3514061	.0177786	-20.67	0.000	.3182326	.3880377
35-	.3279075	.0192892	-18.95	0.000	.2921994	.3679793
40-	.3040547	.0280823	-12.89	0.000	.2537086	.3643916
cal						
2006-	.9191633	.0299236	-2.59	0.010	.862346	.9797241
2011-	.77365	.0272141	-7.30	0.000	.7221085	.8288703
2016-	.7000854	.0269271	-9.27	0.000	.6492495	.7549017
edu						
2	.6595382	.0198464	-13.83	0.000	.6217648	.6996064
3	.3999847	.0160927	-22.78	0.000	.369655	.4328028
9	.9426673	.0588628	-0.95	0.344	.8340786	1.065393
2.marr	1.969131	.0566636	23.55	0.000	1.861146	2.083381
income						
1	.8904782	.0276743	-3.73	0.000	.8378564	.9464048
2	.7387467	.0279926	-7.99	0.000	.6858701	.7956998
3	.5969424	.0273391	-11.27	0.000	.5456934	.6530045
4	.5865313	.0319736	-9.79	0.000	.5270959	.6526687
9	1.075832	.1368712	0.57	0.566	.8384005	1.380503
1.firstpreg	1.075773	.0307442	2.56	0.011	1.017172	1.13775
fland						
2	1.03408	.0492721	0.70	0.482	.9418803	1.135304
3	1.074578	.0383079	2.02	0.044	1.002059	1.152345
9	2.957535	2.958802	1.08	0.278	.4162589	21.01339

```
             |
         psy |
          1  |   3.399919   .1222329    34.04   0.000     3.168593    3.648133
          2  |   3.378726   .0998095    41.21   0.000     3.188658    3.580123
             |
  1.sui_his |    5.45493   .1551247    59.66   0.000     5.159209    5.767602
             |
      season |
          1  |    .928065   .0304429    -2.28   0.023     .8702756    .9896918
          2  |   .9153646   .0298187    -2.71   0.007     .8587476    .9757143
          3  |   .9779027   .0321333    -0.68   0.496     .9169078    1.042955
             |
       _cons |   .0000194   1.43e-06  -147.18   0.000     .0000168    .0000225
      ln(fu) |          1  (exposure)
------------------------------------------------------------------------------
```

Parameter results among fathers:

```
------------------------------------------------------------------------------
          _d |        IRR   Std. err.      z    P>|z|     [95% conf. interval]
-------------+----------------------------------------------------------------
         prd |
          1  |   1.123967   .1052673     1.25   0.212     .9354758    1.350437
          2  |   .6631941   .0600519    -4.54   0.000     .5553473    .7919843
             |
       wksp1 |   .9851347   .0051232    -2.88   0.004     .9751444    .9952273
       wksp2 |   1.026771   .0158103     1.72   0.086     .9962461    1.058231
       wksp3 |   .9171753   .0465793    -1.70   0.089     .8302781    1.013167
             |
 prd#c.wksp1 |
          1  |   .9933073   .0099089    -0.67   0.501     .9740749    1.012919
          2  |   1.033357   .0082761     4.10   0.000     1.017262    1.049706
             |
 prd#c.wksp2 |
```

1	1.025523	.0322232	0.80	0.423	.9642717	1.090664
2	.9338722	.0224923	-2.84	0.005	.8908124	.9790133
prd#c.wksp3						
1	.9299822	.0890639	-0.76	0.448	.7708246	1.122002
2	1.22222	.0905475	2.71	0.007	1.057033	1.413221
agec						
20-	.9002272	.0644818	-1.47	0.142	.7823156	1.035911
25-	.6570522	.0468558	-5.89	0.000	.5713455	.7556156
30-	.5351315	.0390127	-8.58	0.000	.4638796	.6173276
35-	.4639474	.0351389	-10.14	0.000	.3999443	.5381929
40-	.4070761	.0319742	-11.44	0.000	.3489932	.4748256
cal						
2006-	1.084122	.0339461	2.58	0.010	1.01959	1.152739
2011-	.9440667	.0317977	-1.71	0.087	.8837569	1.008492
2016-	.8319518	.0305923	-5.00	0.000	.7741017	.8941251
edu						
2	.7136351	.0197424	-12.20	0.000	.6759711	.7533977
3	.4266905	.0160183	-22.69	0.000	.3964224	.4592698
9	.8545655	.0745111	-1.80	0.071	.7203235	1.013825
2.marr	2.193609	.0606933	28.39	0.000	2.07782	2.31585
income						
1	.8862847	.0276778	-3.87	0.000	.833664	.9422268
2	.6814321	.0238612	-10.95	0.000	.6362337	.7298414
3	.6233544	.0233935	-12.59	0.000	.5791496	.6709332
4	.5384921	.0228531	-14.59	0.000	.4955132	.5851989
9	.3970853	.0672081	-5.46	0.000	.2849804	.5532898

```
             |
 1.firstpreg |   .9329881   .0227305    -2.85   0.004     .889484      .97862
             |
       fland |
          2  |   .8811896    .038257    -2.91   0.004     .8093089    .9594546
          3  |   .6911613    .026346    -9.69   0.000     .6414059    .7447764
          9  |   7.03e-06   .0035924    -0.02   0.981            0           .
             |
         psy |
          1  |   2.572873   .1016566    23.92   0.000     2.381149    2.780034
          2  |   2.756162    .075263    37.13   0.000     2.612527    2.907694
             |
   1.sui_his |   6.514169   .1875944    65.07   0.000     6.156675    6.892422
             |
      season |
          1  |   .9958644   .0306658    -0.13   0.893     .9375383    1.057819
          2  |   .9480357    .029496    -1.72   0.086      .891952    1.007646
          3  |    1.00282   .0314009     0.09   0.928     .9431255    1.066292
             |
       _cons |   .0000117   1.15e-06  -114.86   0.000     9.60e-06    .0000141
      ln(fu) |          1  (exposure)
------------------------------------------------------------------------------
```

2) In the analysis of IRRs and IRDs of suicide attempt comparing mothers to fathers, the following Poisson regression was modelled among preconception, antepartum and postpartum period separately.

$\ln(\lambda) = \beta_0+\beta_1*1.mot+ \beta_2*wksp1+ \beta_3*wksp2+\beta_4*wksp3+ \beta_5*1.mot*wksp1+ \beta_6*1.mot*wksp2+ \beta_7*1.mot*wksp3+ \beta_8*1.agec+ \beta_9*2.agec+ \beta_{10}*3.agec+ \beta_{11}*4.agec+ \beta_{12}*5.agec+ \beta_{13}*1.cal+ \beta_{14}*2.cal+ \beta_{15}*3.cal+ \beta_{16}*2.edu+ \beta_{17}*3.edu+ \beta_{18}*9.edu+ \beta_{19}*2.marr+ \beta_{20}*1.income+ \beta_{21}*2.income+ \beta_{22}*3.income+ \beta_{23}*4.income+ \beta_{24}*9.income+ \beta_{25}*1.firstpreg+ \beta_{26}*2.fland+ \beta_{27}*3.fland+ \beta_{28}*9.fland+ \beta_{29}*1.psy+ \beta_{30}*2.psy+ \beta_{31}*1.sui\_his+ \beta_{32}*1.season+ \beta_{33}*2.season+ \beta_{34}*3.season$

Parameter results in the preconception period:

_d	IRR	Std. err.	z	P>|z|	[95% conf.	interval]
1.mot	1.061776	.0809455	0.79	0.432	.9144097	1.232892
wksp1	.9852645	.0051239	-2.85	0.004	.9752729	.9953585
wksp2	1.026688	.0158091	1.71	0.087	.9961657	1.058146
wksp3	.9171111	.0465766	-1.70	0.088	.8302189	1.013097
mot#c.wksp1						
1	.99403	.0066683	-0.89	0.372	.9810459	1.007186
mot#c.wksp2						
1	1.016324	.0202403	0.81	0.416	.9774185	1.056779
mot#c.wksp3						
1	.9512945	.06254	-0.76	0.448	.8362869	1.082118
agec						
20-	.6384565	.0266954	-10.73	0.000	.588221	.6929822
25-	.4111115	.0186036	-19.64	0.000	.3762192	.4492398
30-	.3441921	.0172161	-21.32	0.000	.3120503	.3796446
35-	.3112973	.0181217	-20.05	0.000	.2777307	.3489208
40-	.2644504	.0199694	-17.61	0.000	.2280697	.3066345
cal						
2006-	.9647363	.0309309	-1.12	0.263	.9059783	1.027305
2011-	.8850658	.0309504	-3.49	0.000	.8264363	.9478546
2016-	.8255758	.0324725	-4.87	0.000	.7643222	.8917383
edu						
2	.6483416	.0189294	-14.84	0.000	.6122823	.6865245
3	.3395092	.0139809	-26.23	0.000	.3131837	.3680474

9	.8383447	.0584191	-2.53	0.011	.7313202	.9610317
2.marr	1.957365	.0574034	22.90	0.000	1.848029	2.07317
income						
1	.8512943	.0271427	-5.05	0.000	.7997238	.9061904
2	.6866803	.0253543	-10.18	0.000	.6387423	.7382162
3	.6466037	.0259476	-10.87	0.000	.5976959	.6995134
4	.525746	.0251542	-13.44	0.000	.4786856	.5774329
9	.7579137	.0975157	-2.15	0.031	.588981	.9752999
1.firstpreg	1.050941	.0303009	1.72	0.085	.9931994	1.11204
fland						
2	.9305919	.0453013	-1.48	0.139	.8459071	1.023754
3	.8941607	.0341999	-2.92	0.003	.8295809	.9637678
9	2.818529	2.820022	1.04	0.300	.396616	20.02972
psy						
1	3.301794	.1229184	32.09	0.000	3.069458	3.551717
2	3.203627	.0930753	40.07	0.000	3.026299	3.391345
1.sui_his	5.794257	.1674864	60.78	0.000	5.475116	6.132001
season						
1	.94362	.030759	-1.78	0.075	.8852189	1.005874
2	.8893145	.0288637	-3.61	0.000	.8345046	.9477244
3	.956956	.0316081	-1.33	0.183	.8969679	1.020956
_cons	.000019	1.58e-06	-131.14	0.000	.0000162	.0000224
ln(fu)	1 (exposure)					

Parameter results in the antepartum period:

```
------------------------------------------------------------------------------
          _d |        IRR   Std. err.      z    P>|z|     [95% conf. interval]
-------------+----------------------------------------------------------------
       1.mot |  1.083038   .1103401     0.78   0.434     .8869994    1.322405
       wksp1 |  .9785863   .0083303    -2.54   0.011     .9623947    .9950503
       wksp2 |  1.053424   .0288494     1.90   0.057     .9983709    1.111513
       wksp3 |  .8513829   .0691118    -1.98   0.047     .7261525    .9982102
             |
 mot#c.wksp1 |
          1  |  .8857703   .0119456    -8.99   0.000     .8626641    .9094954
             |
 mot#c.wksp2 |
          1  |  1.307817   .0606576     5.79   0.000     1.194174    1.432275
             |
 mot#c.wksp3 |
          1  |  .5212547   .0732183    -4.64   0.000     .3958089    .6864587
             |
        agec |
        20-  |  .8625202   .0831768    -1.53   0.125     .7139766    1.041968
        25-  |  .6569818   .0642423    -4.30   0.000     .5423997    .7957692
        30-  |  .5405463   .0547091    -6.08   0.000     .4432841     .659149
        35-  |  .5043134    .053656    -6.43   0.000     .4093904    .6212456
        40-  |  .4127074   .0480179    -7.61   0.000     .3285536    .5184159
             |
         cal |
      2006-  |  1.133236    .056477     2.51   0.012     1.027778    1.249516
      2011-  |  .9417797   .0499752    -1.13   0.258     .8487517    1.045004
      2016-  |  .7849325   .0452345    -4.20   0.000     .7010981    .8787914
             |
```

edu						
2	.7266177	.0327341	-7.09	0.000	.6652109	.7936931
3	.4702827	.0272804	-13.01	0.000	.4197415	.5269094
9	1.010916	.1149	0.10	0.924	.8090369	1.263171
2.marr	2.069549	.0983063	15.31	0.000	1.88557	2.27148
income						
1	.8954304	.0447055	-2.21	0.027	.8119598	.9874818
2	.7199444	.0402428	-5.88	0.000	.6452369	.8033017
3	.5922915	.0361524	-8.58	0.000	.5255084	.6675616
4	.5936985	.0389001	-7.96	0.000	.5221483	.6750533
9	.387981	.1009567	-3.64	0.000	.2329802	.6461032
1.firstpreg	1.092828	.0417245	2.32	0.020	1.014034	1.177744
fland						
2	.902441	.0622614	-1.49	0.137	.7883018	1.033107
3	.8273933	.0465211	-3.37	0.001	.7410581	.9237869
9	.0000281	.0109978	-0.03	0.979	0	.
psy						
1	2.478756	.1431087	15.72	0.000	2.213555	2.775729
2	2.457031	.1074621	20.55	0.000	2.255184	2.676944
1.sui_his	6.775024	.3018951	42.94	0.000	6.208423	7.393335
season						
1	.9840153	.0486039	-0.33	0.744	.8932193	1.084041
2	.9950405	.0484523	-0.10	0.919	.9044665	1.094685
3	1.018442	.0505154	0.37	0.713	.9240939	1.122423

```
       _cons |   .0000115   1.50e-06   -86.91   0.000     8.87e-06    .0000148
      ln(fu) |          1  (exposure)
------------------------------------------------------------------------------
```

Parameter results in the postpartum period:

```
------------------------------------------------------------------------------
          _d |        IRR   Std. err.      z    P>|z|     [95% conf. interval]
-------------+----------------------------------------------------------------
       1.mot |    .214745    .027817   -11.88   0.000     .1665952    .2768112
       wksp1 |   1.018004   .0062007     2.93   0.003     1.005923     1.03023
       wksp2 |   .9587975   .0177572    -2.27   0.023     .9246181    .9942403
       wksp3 |   1.121202   .0604768     2.12   0.034      1.00872    1.246227
             |
 mot#c.wksp1 |
          1  |   1.035885   .0114306     3.20   0.001     1.013722    1.058532
             |
 mot#c.wksp2 |
          1  |   .9645145   .0308226    -1.13   0.258     .9059563    1.026858
             |
 mot#c.wksp3 |
          1  |   1.047851   .0956652     0.51   0.609      .876169    1.253174
             |
        agec |
        20-  |   .7677135   .0709682    -2.86   0.004     .6404914    .9202059
        25-  |   .5573252   .0518075    -6.29   0.000     .4644973    .6687044
        30-  |    .483911     .04596    -7.64   0.000     .4017183    .5829204
        35-  |   .4045957   .0400075    -9.15   0.000     .3333128    .4911232
        40-  |   .3672069   .0381747    -9.64   0.000     .2995161    .4501959
             |
         cal |
      2006-  |   .9698605   .0400145    -0.74   0.458     .8945206    1.051546
```

```
      2011-  |  .7459754   .0330291    -6.62   0.000      .683969    .8136032
      2016-  |  .6697648   .0311854    -8.61   0.000     .6113486    .7337629
             |
        edu  |
          2  |  .7356329   .0269128    -8.39   0.000     .6847316    .7903181
          3  |  .4914866   .0235385   -14.83   0.000     .4474511    .5398558
          9  |  1.092532   .1049127     0.92   0.357     .9050986    1.318781
             |
     2.marr  |  2.363964   .0825529    24.64   0.000     2.207576     2.53143
             |
     income  |
          1  |  .9438576    .036115    -1.51   0.131     .8756626    1.017363
          2  |  .7598277   .0349961    -5.96   0.000     .6942415      .83161
          3  |  .6194015   .0344858    -8.60   0.000      .555368    .6908181
          4  |  .6199103   .0407729    -7.27   0.000     .5449333    .7052032
          9  |  .4607801    .102184    -3.49   0.000     .2983518    .7116375
             |
1.firstpreg  |  .8915715   .0281591    -3.63   0.000     .8380543    .9485064
             |
      fland  |
          2  |  .9788682   .0532884    -0.39   0.695     .8798037    1.089087
          3  |  .8275175   .0375978    -4.17   0.000     .7570129    .9045886
          9  |  .0000258   .0090337    -0.03   0.976     2.8e-303    2.4e+293
             |
        psy  |
          1  |  2.676546   .1300576    20.26   0.000       2.4334    2.943987
          2  |  3.088594   .1096648    31.76   0.000     2.880964    3.311189
             |
  1.sui_his  |  5.830534    .215022    47.81   0.000     5.423969    6.267574
             |
```

season |						
1 |	.9880033	.0392883	-0.30	0.762	.9139241	1.068087
2 |	.9705976	.0395465	-0.73	0.464	.8961019	1.051286
3 |	1.026153	.0411978	0.64	0.520	.9485021	1.110161
|						
_cons |	8.72e-06	1.06e-06	-96.21	0.000	6.87e-06	.0000111
ln(fu) |	1 (exposure)					

Figure S1 Flow chart

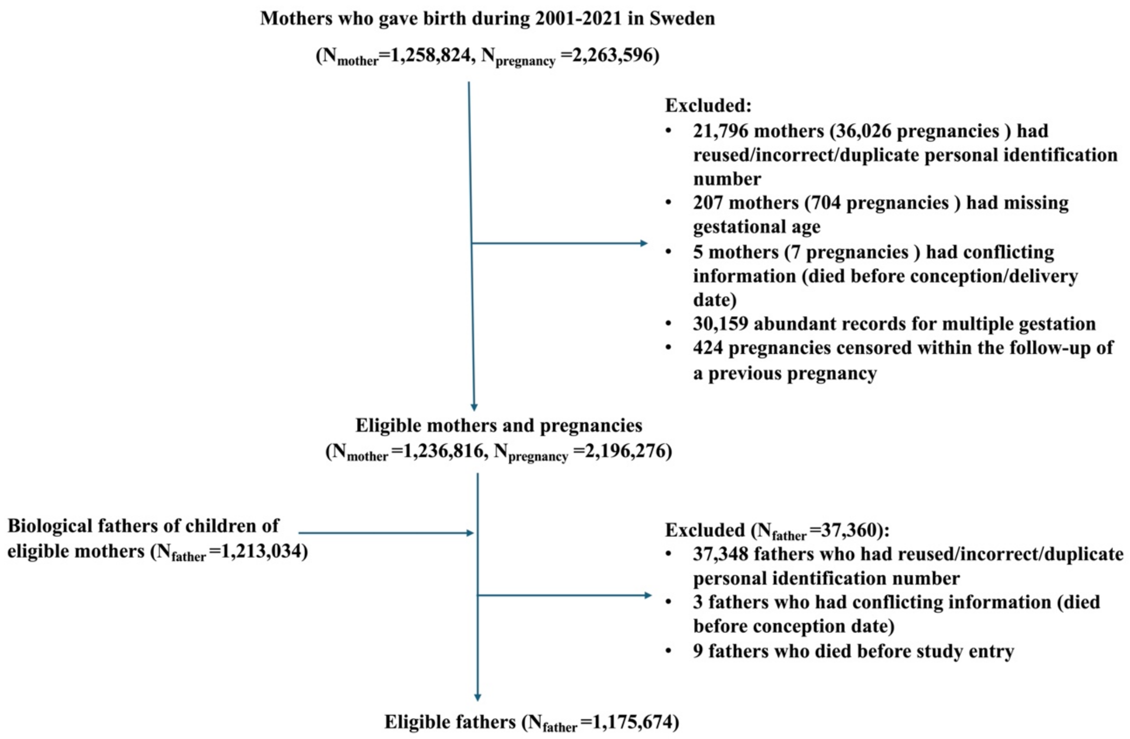

Figure S2 Incidence rate of parental suicide attempt before, during, and after pregnancy, by age at start of each period

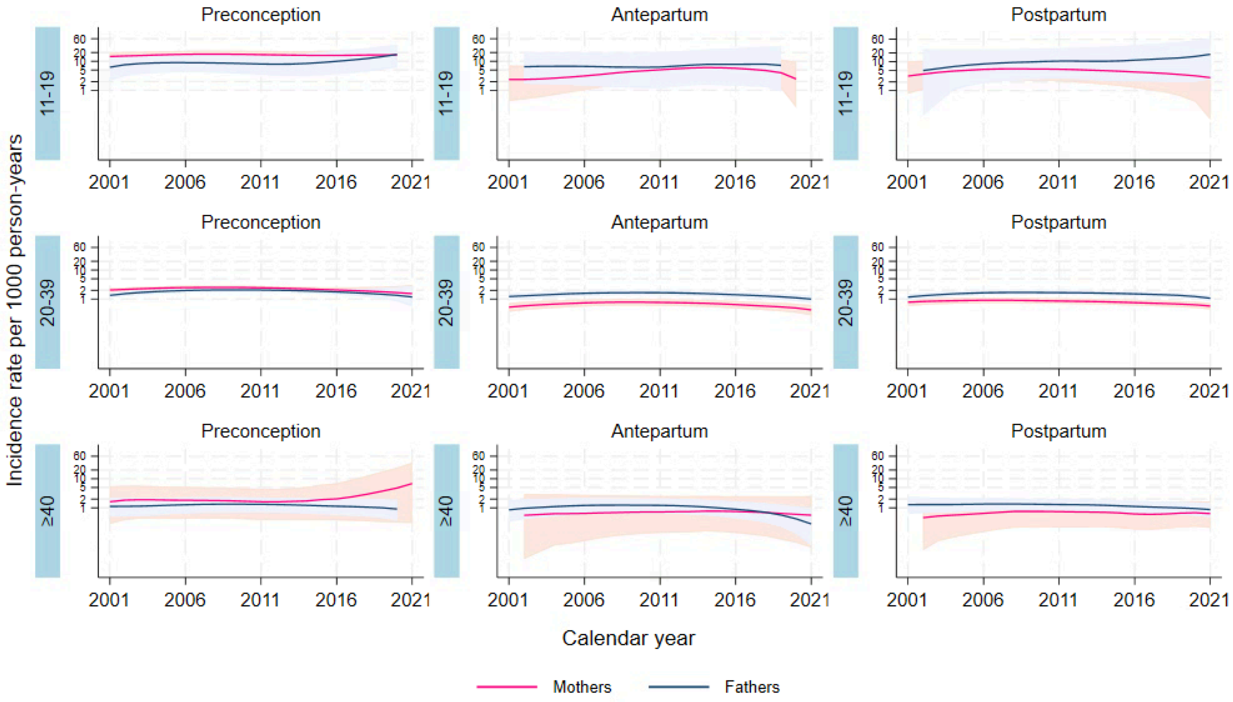

The analyses are based on aggregated data from 2,196,276 pregnancies (1,236,816 mothers and 1,175,674 fathers). Locally Weighted Scatterplot Smoothing (bandwidth=0.8) was used to smooth the lines. The shaded areas indicate 95% confidence interval. Note that a logarithmic scale was used.

Figure S3 Standardized incidence rate of parental suicide attempt before, during, and after pregnancy, by time-varying depressive disorders

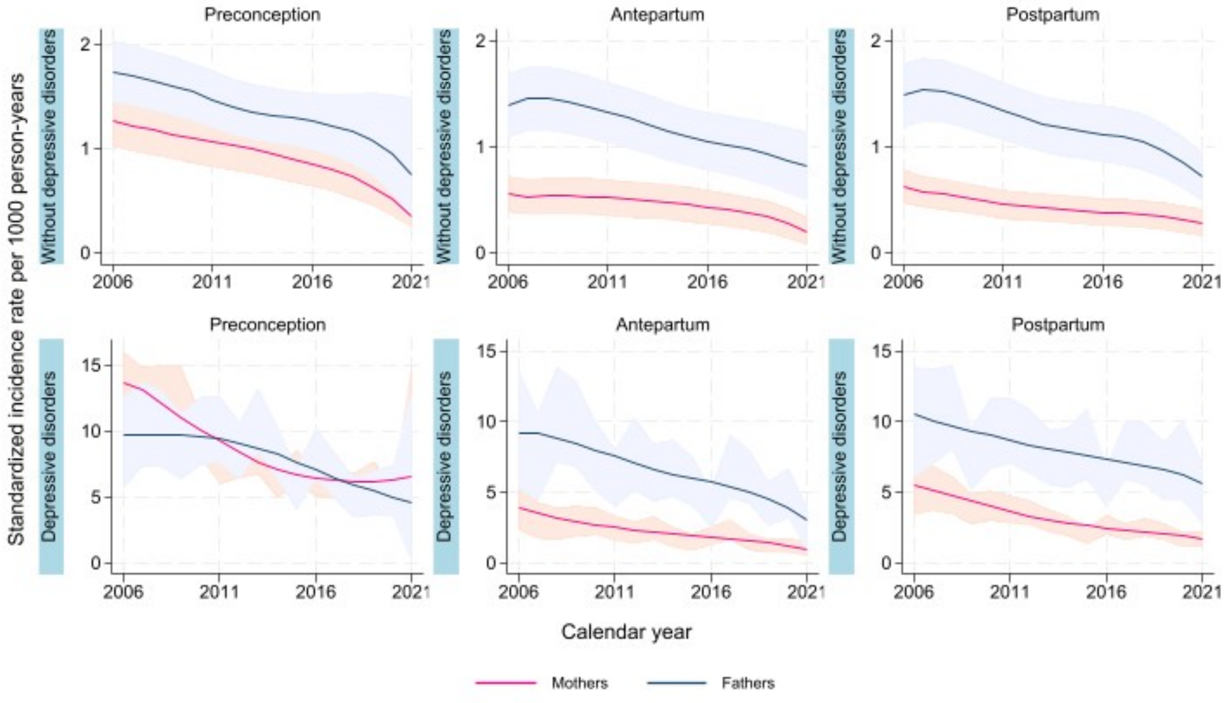

The analyses are based on aggregated data from 1,825,285 pregnancies (1,067,164 mothers and 1,010,422 fathers). Standardized incidence rate was standardized by distribution of age group of the accumulated person-years during follow-up. Locally Weighted Scatterplot Smoothing (bandwidth=0.8) was used to smooth the lines. The shaded areas indicate 95% confidence interval. Note that we started follow-up from 2006 since the Prescribed Drug Registry was incepted in July 2005.

Figure S4 Standardized incidence rate of parental suicide attempt before, during, and after pregnancy, by history of suicide attempt

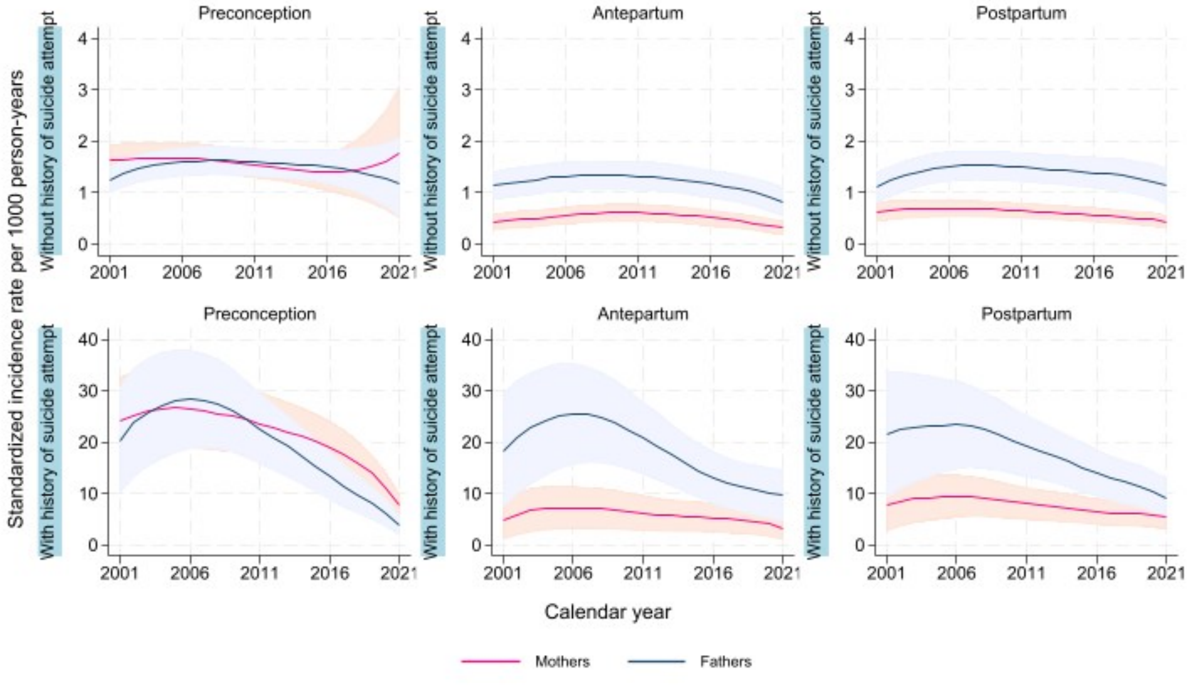

The analyses are based on aggregated data from 2,196,276 pregnancies (1,236,816 mothers and 1,175,674 fathers). The incidence rate was standardized by the distribution of age group of the accumulated person-years during follow-up. Locally Weighted Scatterplot Smoothing (bandwidth=0.8) was used to smooth the lines. The shaded areas indicate 95% confidence interval.

Figure S5 Standardized incidence rate of parental suicide attempt before, during, and after pregnancy, by week and time-varying depressive disorders

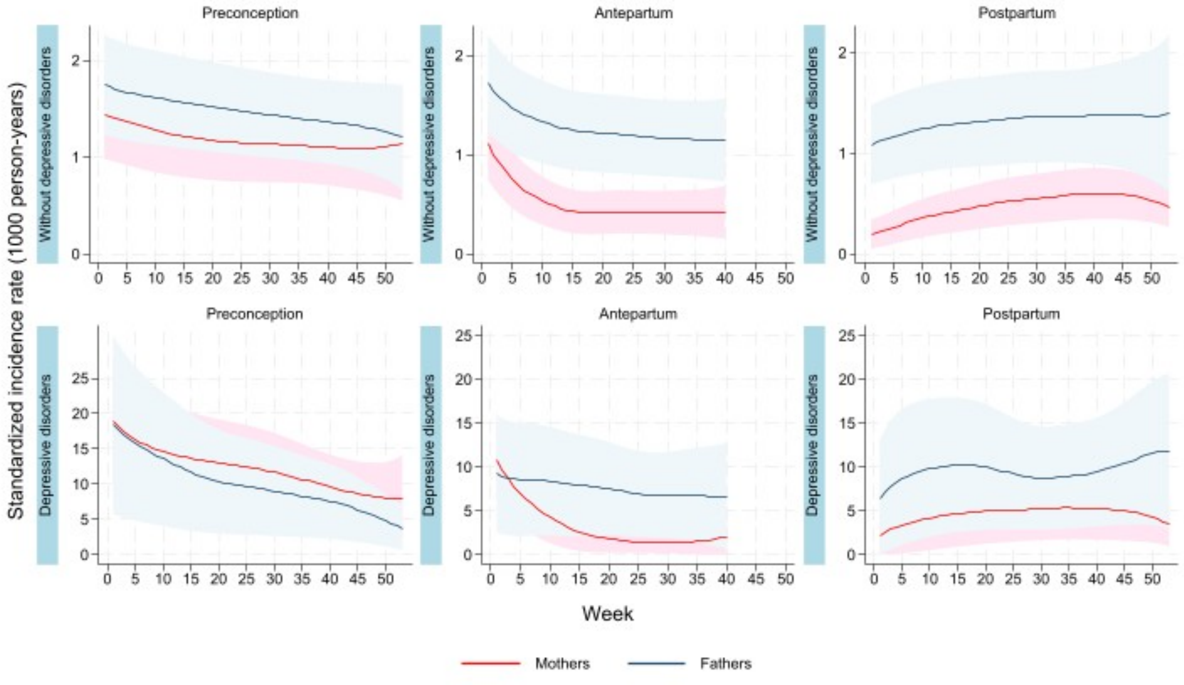

The follow-up during pregnancy was censored at week 40 due to few subsequent cases.

The analyses are based on aggregated data from 2,196,276 pregnancies (1,236,816 mothers and 1,175,674 fathers). The incidence rates were standardized by the distribution of age group and calendar period of the accumulated person-days during follow-up. Locally Weighted Scatterplot Smoothing (bandwidth=0.8) was used to smooth the lines. The shaded areas indicate 95% confidence interval.

Figure S6 Standardized incidence rate of parental suicide attempt before, during, and after pregnancy, by week and history of suicide attempt

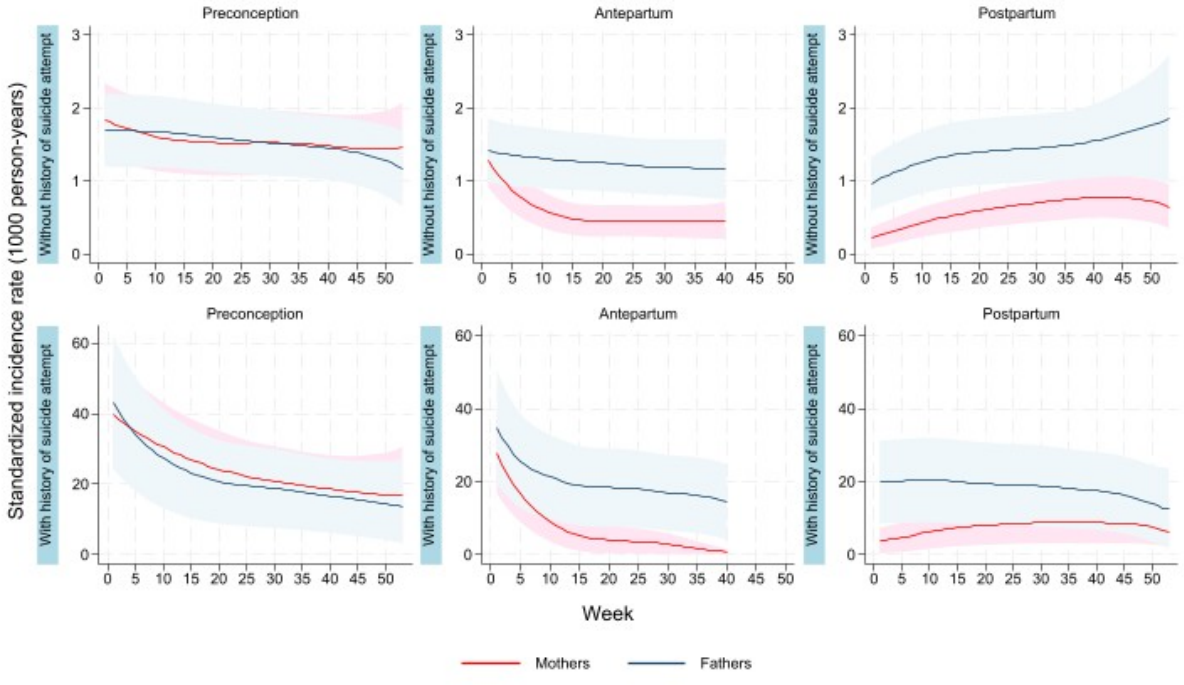

The follow-up during pregnancy was censored at week 40 due to few subsequent cases.

The analyses are based on aggregated data from 2,196,276 pregnancies (1,236,816 mothers and 1,175,674 fathers). The incidence rates were standardized by distribution of age group and calendar period of the accumulated person-days during follow-up in each period. Locally Weighted Scatterplot Smoothing (bandwidth=0.8) was used to smooth the lines. The shaded areas indicate 95% confidence interval.

Figure S7 Incidence rate ratio of parental suicide attempt during and after pregnancy when comparing with the corresponding week before pregnancy, by time-varying depressive disorders

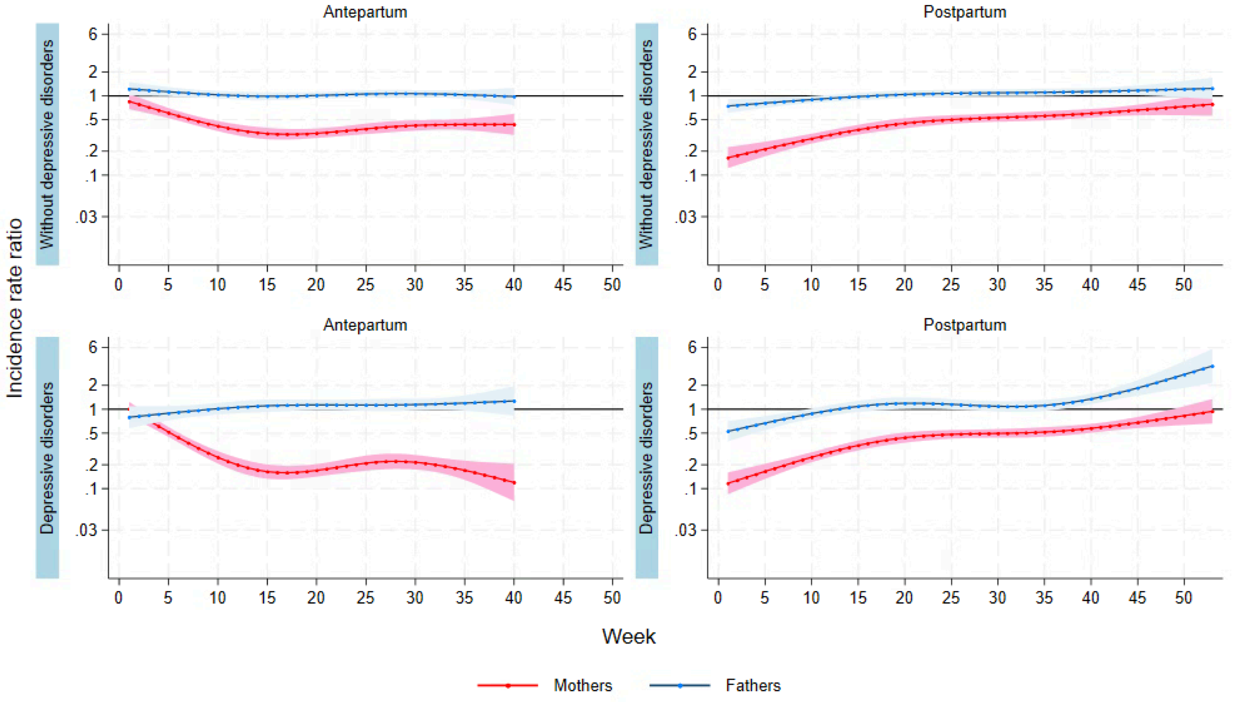

The follow-up during pregnancy was censored at week 40 due to few subsequent cases.

The analyses are based on aggregated data from 2,196,276 pregnancies (1,236,816 mothers and 1,175,674 fathers). A restricted cubic spline with 4 knots, placed at 5th percentile, 35th percentile, 65th percentile and 95th percentile of distribution of weeks on the relevant x axes, were used to estimate the incidence rate ratios. The shaded areas indicate 95% confidence interval. The incidence rate ratios were estimated based on Poisson regression, and adjusted for country of birth, age, calendar year, education level, civil status, category of income, primiparity, history of psychiatric disorders, history of suicide attempt and season, all derived at the start of each period. Note that a logarithmic scale was used.

Figure S8 Incidence rate ratio of parental suicide attempt during and after pregnancy when comparing with the corresponding week before pregnancy, by history of suicide attempt

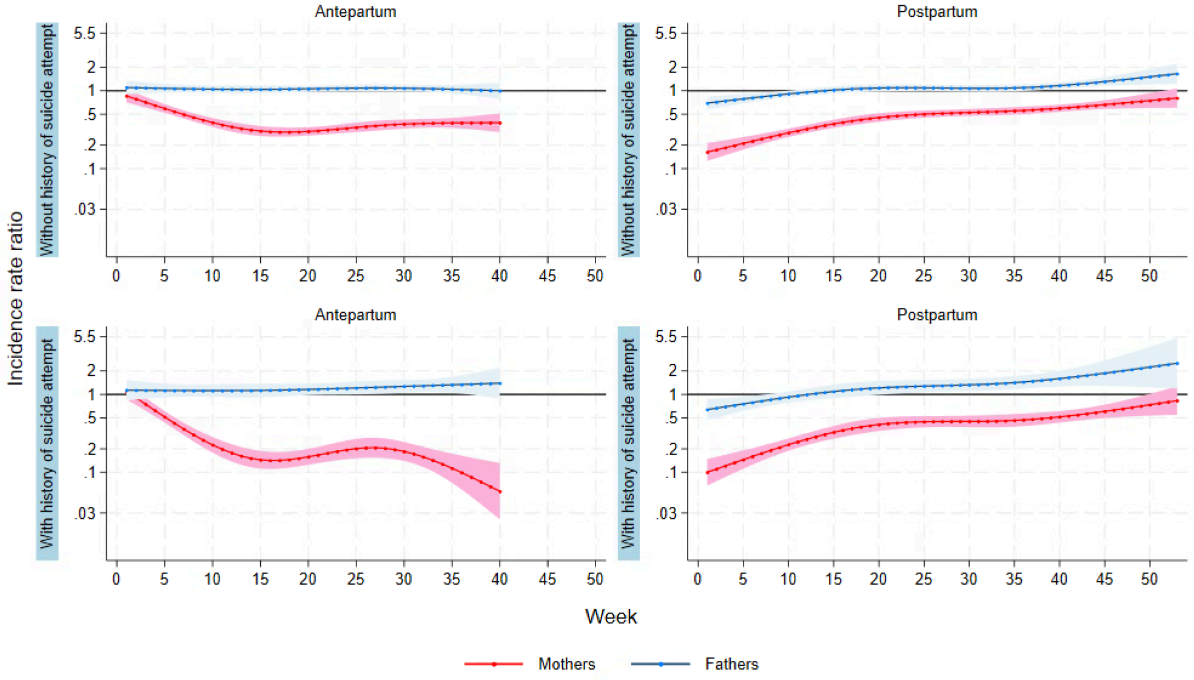

The follow-up during pregnancy was censored at week 40 due to few subsequent cases.

The analyses are based on aggregated data from 2,196,276 pregnancies (1,236,816 mothers and 1,175,674 fathers). A restricted cubic spline with 4 knots, placed at 5th percentile, 35th percentile, 65th percentile and 95th percentile of distribution of weeks on the relevant x axes, were used to estimate the incidence rate ratios. The shaded areas indicate 95% confidence interval. The incidence rate ratios were estimated based on Poisson regression, and adjusted for country of birth, age, calendar year, education level, civil status, category of income, primiparity, history of psychiatric disorders, history of suicide attempt and season, all derived at the start of each period. Note that a logarithmic scale was used.

Figure S9 Incidence rate ratio of suicide attempt among mothers compared to the corresponding week among fathers, by suicidal method

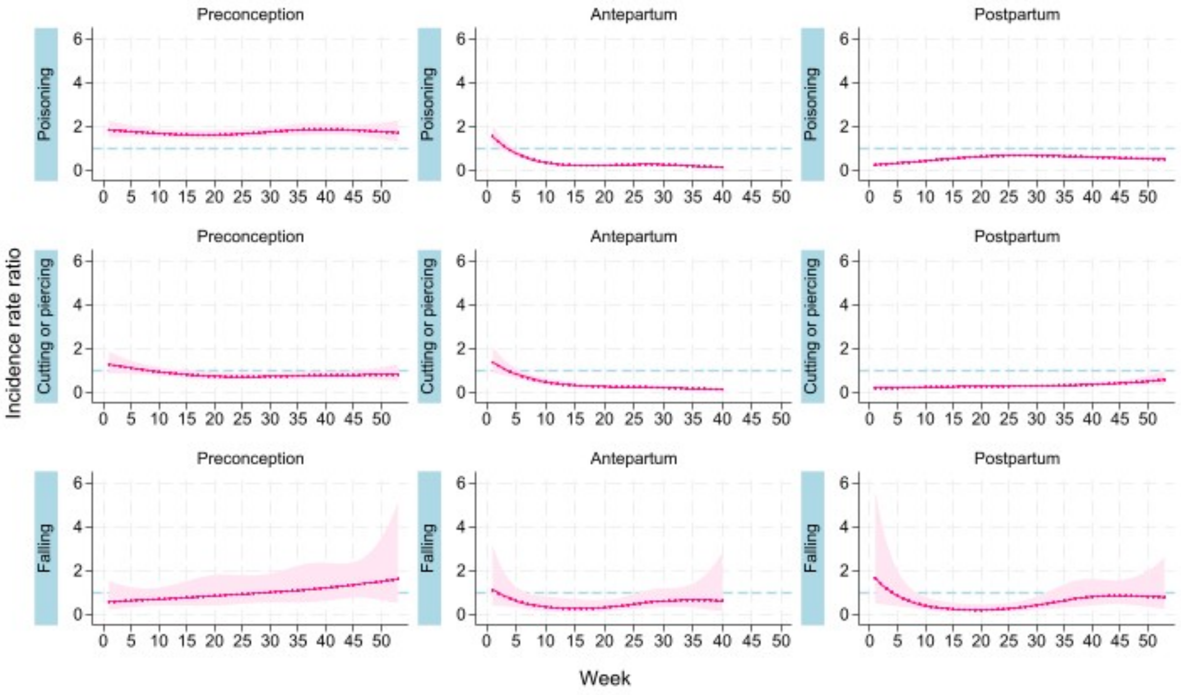

The follow-up during pregnancy was censored at week 40 due to few subsequent cases.

The analyses are based on aggregated data from 2,196,276 pregnancies (1,236,816 mothers and 1,175,674 fathers). A restricted cubic spline with 4 knots, placed at 5th percentile, 35th percentile, 65th percentile and 95th percentile of distribution of weeks on the relevant x axes, were used to estimate the incidence rate ratios. The shaded areas indicate 95% confidence interval. The incidence rate ratios were estimated based on Poisson regression, and adjusted for country of birth, age, calendar year, education level, civil status, category of income, primiparity, history of psychiatric disorders, history of suicide attempt and season, all derived at the start of each period.

Figure S10 Incidence rate ratio of parental suicide attempt during and after pregnancy when compared with average incidence rate before pregnancy

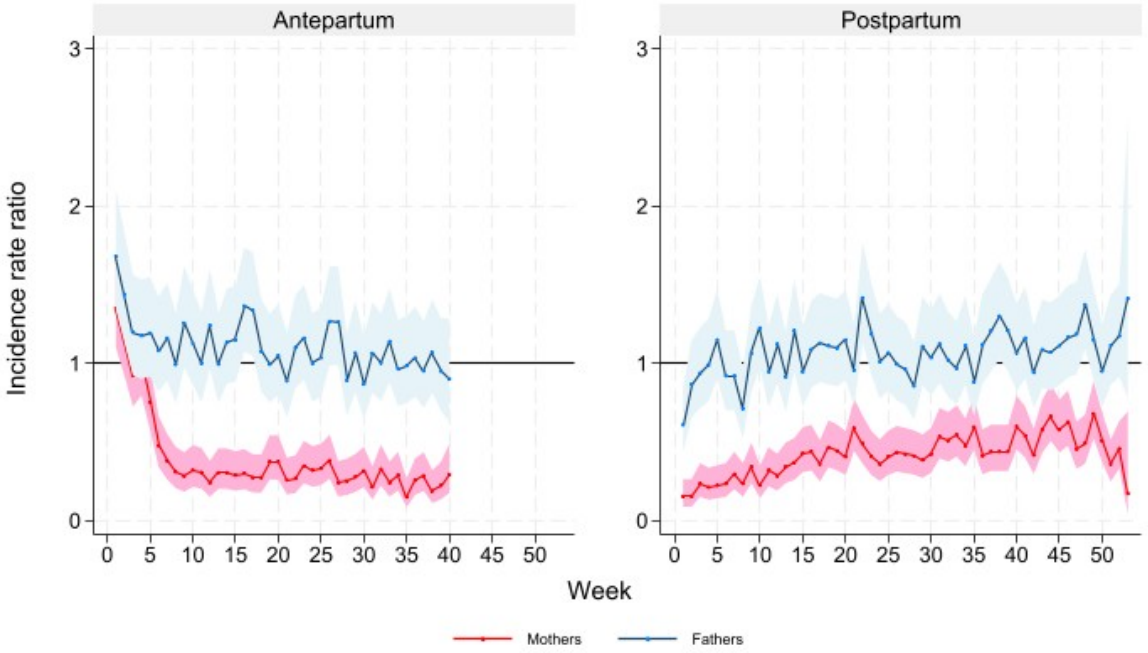

The analyses are based on aggregated data from 2,196,276 pregnancies (1,236,816 mothers and 1,175,674 fathers). The incidence rate ratios were estimated based on Poisson regression, and adjusted for country of birth, age, calendar year, education level, civil status, category of income, primiparity, history of psychiatric disorders, and history of suicide attempt, all derived at the start of each period. The shaded areas indicate 95% confidence interval.

Figure S11 Standardized incidence rate of parental suicide attempt before, during, and after pregnancy by week, after excluding week 53 from the analysis

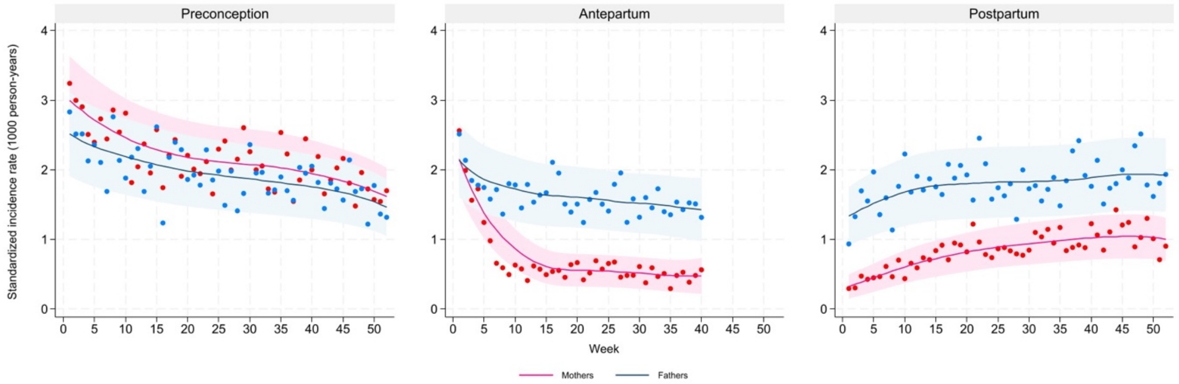

The follow-up during pregnancy was censored at week 40 due to few subsequent events.

The analyses are based on aggregated data from 2,196,276 pregnancies (1,236,816 mothers and 1,175,674 fathers). Incidence rates were standardized by distribution of age group and calendar period of the accumulated person-days during follow-up. Locally Weighted Scatterplot Smoothing (bandwidth=0.8) was used to estimate the trend (solid line), while the dots indicate the weekly incidence rates. The shaded areas indicate 95% confidence interval.

Figure S12 Incidence rate ratio of parental suicide attempt during and after pregnancy when compared with the corresponding week before pregnancy, after excluding week 53 from the analysis

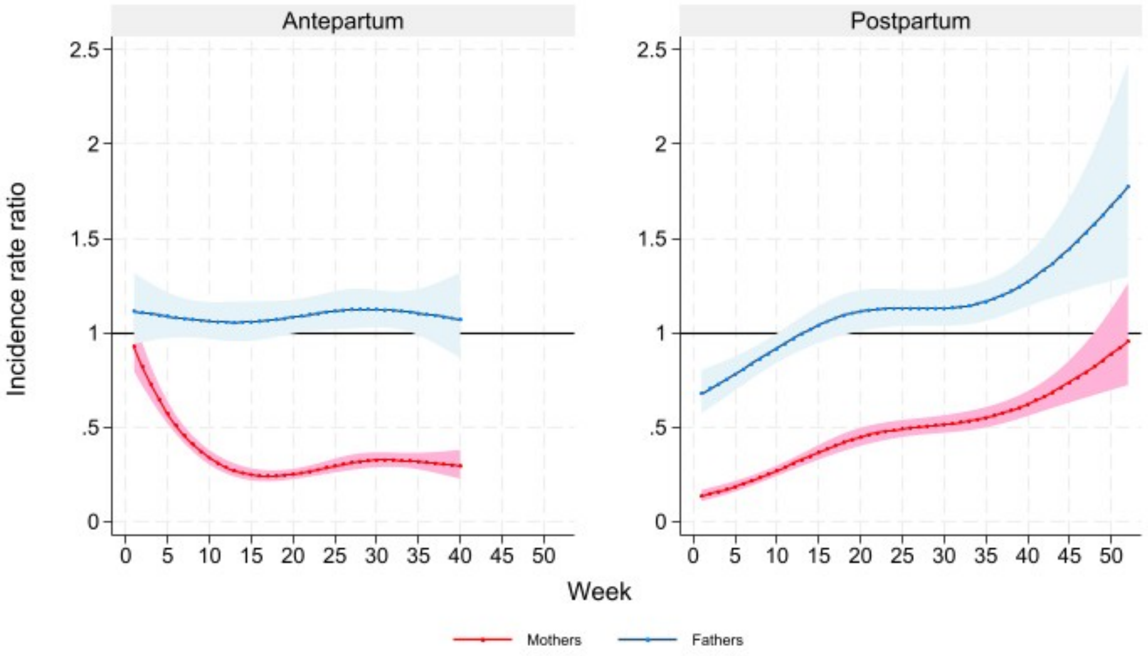

The follow-up during pregnancy was censored at week 40 due to few subsequent events.

The analyses are based on aggregated data from 2,196,276 pregnancies (1,236,816 mothers and 1,175,674 fathers). A restricted cubic spline with 4 knots, placed at 5th percentile, 35th percentile, 65th percentile and 95th percentile of distribution of weeks on the relevant x axes, was used to estimate the incidence rate ratios. The incidence rate ratios were estimated based on Poisson regression, and adjusted for country of birth, age, calendar year, education level, civil status, category of income, primiparity, history of psychiatric disorders, history of suicide attempt and season, all derived at the start of each period. The shaded areas indicate 95% confidence interval.

Figure S13 Incidence rate ratio of suicide attempt among mothers when compared with the corresponding week among fathers in preconception and postpartum period, after excluding week 53

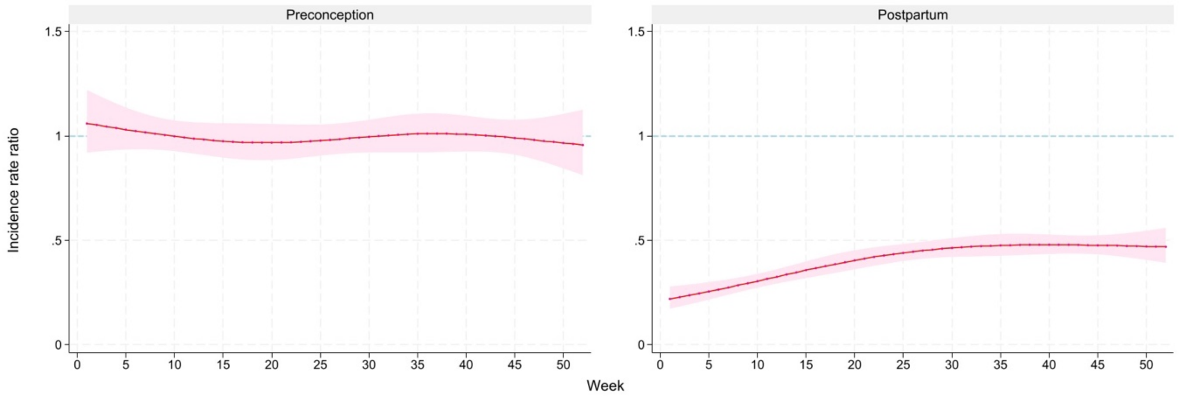

The analyses are based on aggregated data from 2,196,276 pregnancies (1,236,816 mothers and 1,175,674 fathers). A restricted cubic spline with 4 knots, placed at 5th percentile, 35th percentile, 65th percentile and 95th percentile of distribution of weeks on the relevant x axes, was used to estimate the incidence rate ratios. The incidence rate ratios were estimated based on Poisson regression, and adjusted for country of birth, age, calendar year, education level, civil status, category of income, primiparity, history of psychiatric disorders, history of suicide attempt and season, all derived at the start of each period. The shaded areas indicate 95% confidence interval.
