# Supplementary material for: Sex difference in parental risk of suicide attempt during and after pregnancy in Sweden
Source: Nat Hum Behav. 2025 Sep 25;10(1):101–10. doi: 10.1038/s41562-025-02311-5 (PMC12846918; doi:10.1038/s41562-025-02311-5)
Supplement: Supplementary file 2 — Reporting Summary [file 41562_2025_2311_MOESM2_ESM.pdf]

nature portfolio

Corresponding author(s): Yihui Yang, Donghao Lu

Last updated by author(s): Jul 10, 2025

# Reporting Summary

Nature Portfolio wishes to improve the reproducibility of the work that we publish. This form provides structure for consistency and transparency in reporting. For further information on Nature Portfolio policies, see our Editorial Policies and the Editorial Policy Checklist.

## Statistics

For all statistical analyses, confirm that the following items are present in the figure legend, table legend, main text, or Methods section.

| n/a | Confirmed | |
|---|---|---|
| ☐ | ☒ | The exact sample size (*n*) for each experimental group/condition, given as a discrete number and unit of measurement |
| ☐ | ☒ | A statement on whether measurements were taken from distinct samples or whether the same sample was measured repeatedly |
| ☐ | ☒ | The statistical test(s) used AND whether they are one- or two-sided<br>*Only common tests should be described solely by name; describe more complex techniques in the Methods section.* |
| ☐ | ☒ | A description of all covariates tested |
| ☒ | ☐ | A description of any assumptions or corrections, such as tests of normality and adjustment for multiple comparisons |
| ☐ | ☒ | A full description of the statistical parameters including central tendency (e.g. means) or other basic estimates (e.g. regression coefficient) AND variation (e.g. standard deviation) or associated estimates of uncertainty (e.g. confidence intervals) |
| ☐ | ☒ | For null hypothesis testing, the test statistic (e.g. *F*, *t*, *r*) with confidence intervals, effect sizes, degrees of freedom and *P* value noted<br>*Give P values as exact values whenever suitable.* |
| ☒ | ☐ | For Bayesian analysis, information on the choice of priors and Markov chain Monte Carlo settings |
| ☒ | ☐ | For hierarchical and complex designs, identification of the appropriate level for tests and full reporting of outcomes |
| ☒ | ☐ | Estimates of effect sizes (e.g. Cohen's *d*, Pearson's *r*), indicating how they were calculated |

*Our web collection on statistics for biologists contains articles on many of the points above.*

## Software and code

Policy information about availability of computer code

| | |
|---|---|
| Data collection | No software was used in data collection. |
| Data analysis | Data were cleaned using SAS, version 9.4 (SAS institute, Cary, NC) and analyzed using Stata 18.0 (STATA, College Station, TX). Analysis coding is available at Github (https://github.com/yihuiyang2/ParentalSuicide). |

For manuscripts utilizing custom algorithms or software that are central to the research but not yet described in published literature, software must be made available to editors and reviewers. We strongly encourage code deposition in a community repository (e.g. GitHub). See the Nature Portfolio guidelines for submitting code & software for further information.

## Data

Policy information about availability of data

All manuscripts must include a data availability statement. This statement should provide the following information, where applicable:

- Accession codes, unique identifiers, or web links for publicly available datasets
- A description of any restrictions on data availability
- For clinical datasets or third party data, please ensure that the statement adheres to our policy

The Public Access to Information and Secrecy Act in Sweden prohibits individual-level data being publicly available. Researchers who are interested in replicating this study can apply for individual-level data from Total Population Register, Multi-Generation Register, and Longitudinal Integration Database for Health Insurance and Labor Market Studies through Statistics Sweden (https://www.scb.se/en/services/ordering-data-and-statistics/ordering-microdata/). Data on patient health

from Swedish Medical Birth Register, National Patient Register, and Prescribed Drug Register can be applied for through Socialstyrelsen (https://www.socialstyrelsen.se/en/statistics-and-data/registers/).

## Research involving human participants, their data, or biological material

Policy information about studies with human participants or human data. See also policy information about sex, gender (identity/presentation), and sexual orientation and race, ethnicity and racism.

| | |
|---|---|
| Reporting on sex and gender | This study analyzed sex-specific incidence rate of suicide attempt. Sex was determined based on Swedish Medical Birth Register and Total Population Register. |
| Reporting on race, ethnicity, or other socially relevant groupings | We collected information on participants' country of birth from Swedish Total Population Register, as a proxy of potential differential health care-seeking behaviour. |
| Population characteristics | In the study sample, mothers were younger, had a higher educational attainment yet a lower income, than the fathers (Table 1). In addition, mothers were more likely to have a history of depression and other psychiatric disorders or a history of suicide attempt. |
| Recruitment | We identified mothers from Swedish Medical Birth Register (MBR), which includes antenatal and obstetric records on 98% of all births in Sweden. Fathers were mainly identified from Multi-Generation Register, which provides information on familial linkages for individuals born since 1932 in Sweden. Because fathers were not reported for 2% of children in the MGR, we complemented the identification of fathers as partners of women who reported as cohabitating with “the-father-to-be” at their first antenatal visit, based on the MBR. Selection bias could emerge when participation is related to both exposure and outcome. In our study, exposure is sex and period, which should not be affected by participation. Therefore, the selection bias should be minimized in our study. |
| Ethics oversight | Swedish Ethical Review Authority |

Note that full information on the approval of the study protocol must also be provided in the manuscript.

# Field-specific reporting

Please select the one below that is the best fit for your research. If you are not sure, read the appropriate sections before making your selection.

☒ Life sciences ☐ Behavioural & social sciences ☐ Ecological, evolutionary & environmental sciences

For a reference copy of the document with all sections, see nature.com/documents/nr-reporting-summary-flat.pdf

# Life sciences study design

All studies must disclose on these points even when the disclosure is negative.

| | |
|---|---|
| Sample size | No sample size calculation was performed. We determined sample size based on predefined inclusion and exclusion criteria. Specifically, based on the MBR, we first identified 1,258,824 women and their 2,263,596 pregnancies during 2001-2021 in Sweden. After excluding women who had an invalid personal identification number, missing information on length of gestation, conflicting information (e.g., died before start of pregnancy or delivery date), and erroneous records, 1,236,816 women and 2,196,276 pregnancies remained in the analysis (Figure S1). We then identified fathers based on the Multi-Generation Register (MGR) . Because fathers were not reported for 2% of children in the MGR, we complemented the identification of fathers as partners of women who reported as cohabitating with “the-father-to-be” at their first antenatal visit, based on the MBR. Among the 1,213,034 fathers identified, we excluded those who had invalid personal identification numbers or conflicting information, leaving 1,175,674 fathers in the analysis (Figure S1). |
| Data exclusions | Among 1,258,824 mothers who gave birth during 2001-2021 in Sweden, we excluded women who had an invalid personal identification number, missing information on length of gestation or conflicting information (e.g., died before start of pregnancy or delivery date), leaving 1,236,816 mothers in the analysis. Among the 1,213,034 fathers identified, we excluded those who had invalid personal identification numbers or conflicting information, leaving 1,175,674 fathers in the analysis. |
| Replication | We have rerun the analysis coding several times, and the results consistently matched to what we presented in the manuscript. |
| Randomization | This is an observational study and no randomization was performed. |
| Blinding | This is an observational study and no randomization was performed. |

# Reporting for specific materials, systems and methods

We require information from authors about some types of materials, experimental systems and methods used in many studies. Here, indicate whether each material, system or method listed is relevant to your study. If you are not sure if a list item applies to your research, read the appropriate section before selecting a response.

## Materials & experimental systems

| n/a | Involved in the study |
|---|---|
| ☒ | ☐ Antibodies |
| ☒ | ☐ Eukaryotic cell lines |
| ☒ | ☐ Palaeontology and archaeology |
| ☒ | ☐ Animals and other organisms |
| ☒ | ☐ Clinical data |
| ☒ | ☐ Dual use research of concern |
| ☒ | ☐ Plants |

## Methods

| n/a | Involved in the study |
|---|---|
| ☒ | ☐ ChIP-seq |
| ☒ | ☐ Flow cytometry |
| ☒ | ☐ MRI-based neuroimaging |

# Plants

Seed stocks

*Report on the source of all seed stocks or other plant material used. If applicable, state the seed stock centre and catalogue number. If plant specimens were collected from the field, describe the collection location, date and sampling procedures.*

Novel plant genotypes

*Describe the methods by which all novel plant genotypes were produced. This includes those generated by transgenic approaches, gene editing, chemical/radiation-based mutagenesis and hybridization. For transgenic lines, describe the transformation method, the number of independent lines analyzed and the generation upon which experiments were performed. For gene-edited lines, describe the editor used, the endogenous sequence targeted for editing, the targeting guide RNA sequence (if applicable) and how the editor was applied.*

Authentication

*Describe any authentication procedures for each seed stock used or novel genotype generated. Describe any experiments used to assess the effect of a mutation and, where applicable, how potential secondary effects (e.g. second site T-DNA insertions, mosiacism, off-target gene editing) were examined.*
